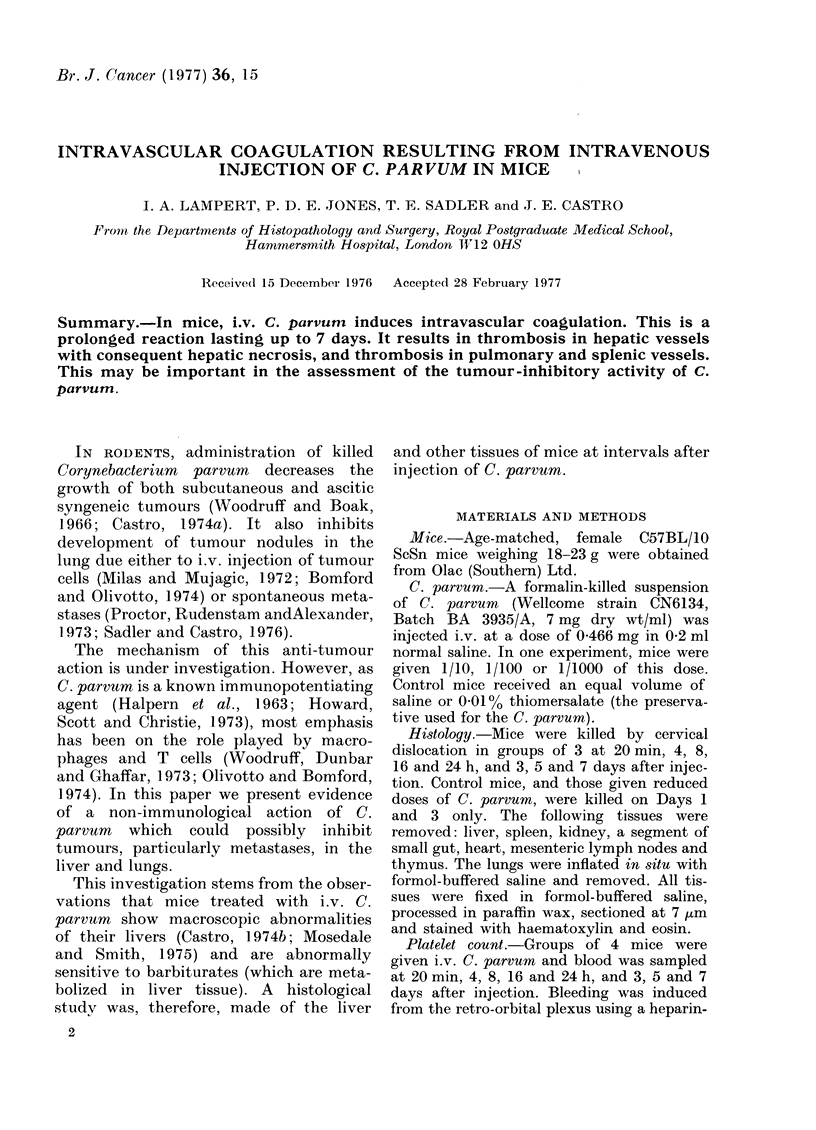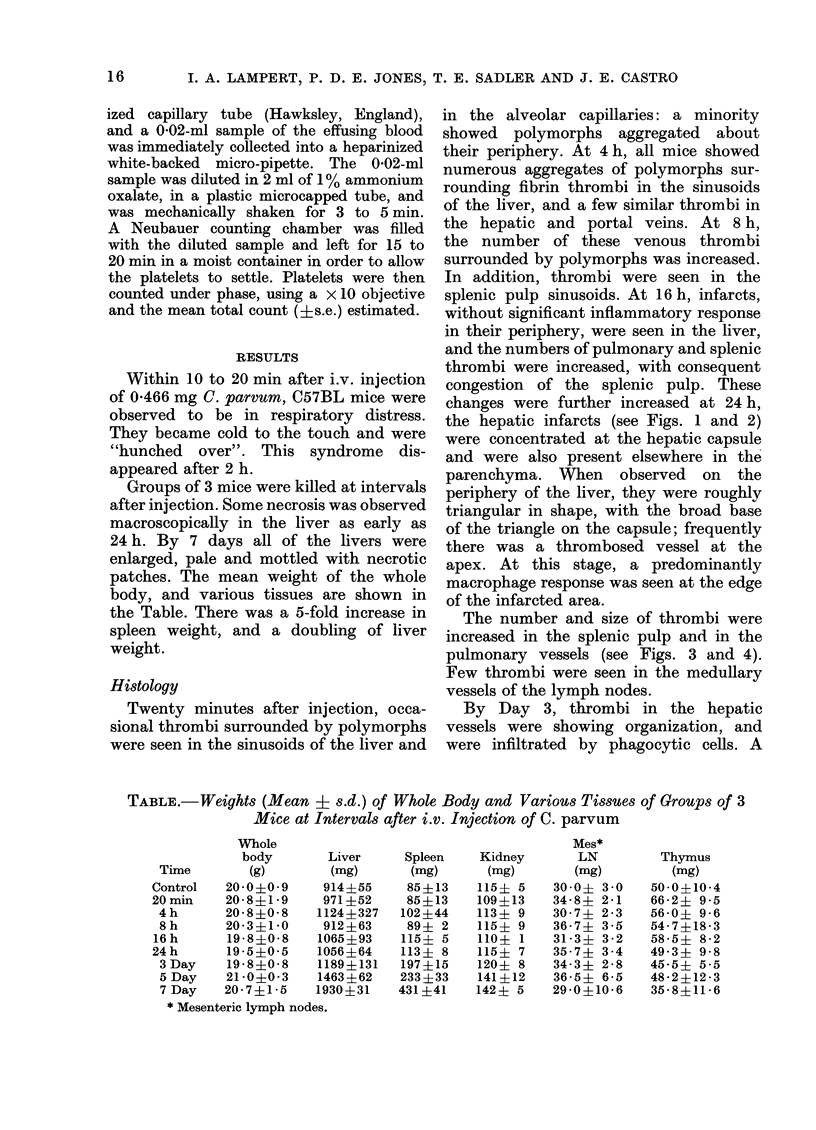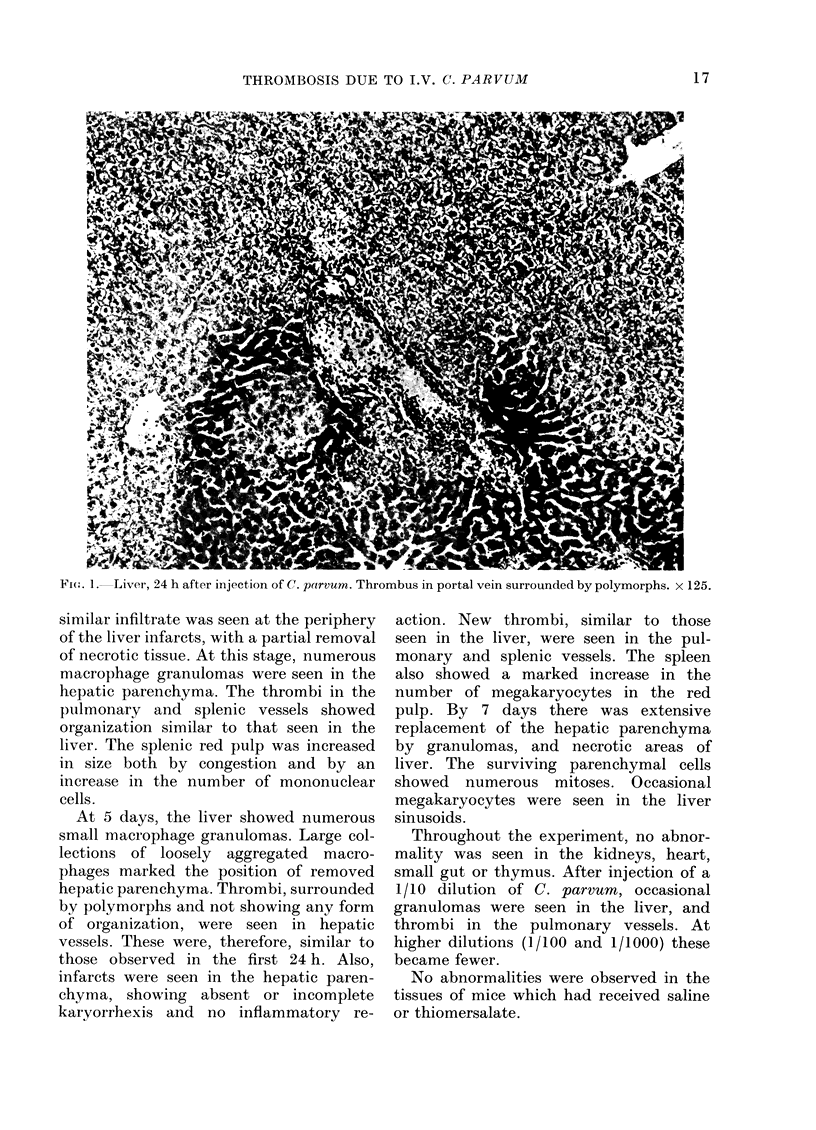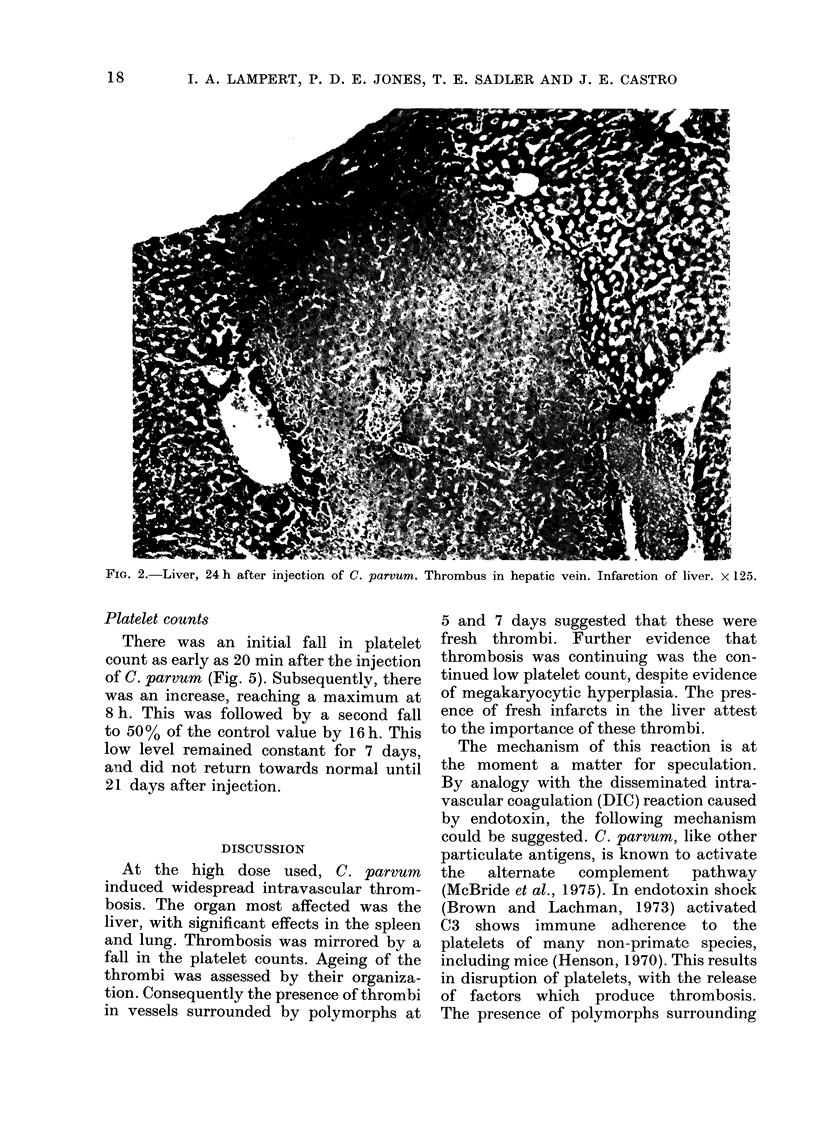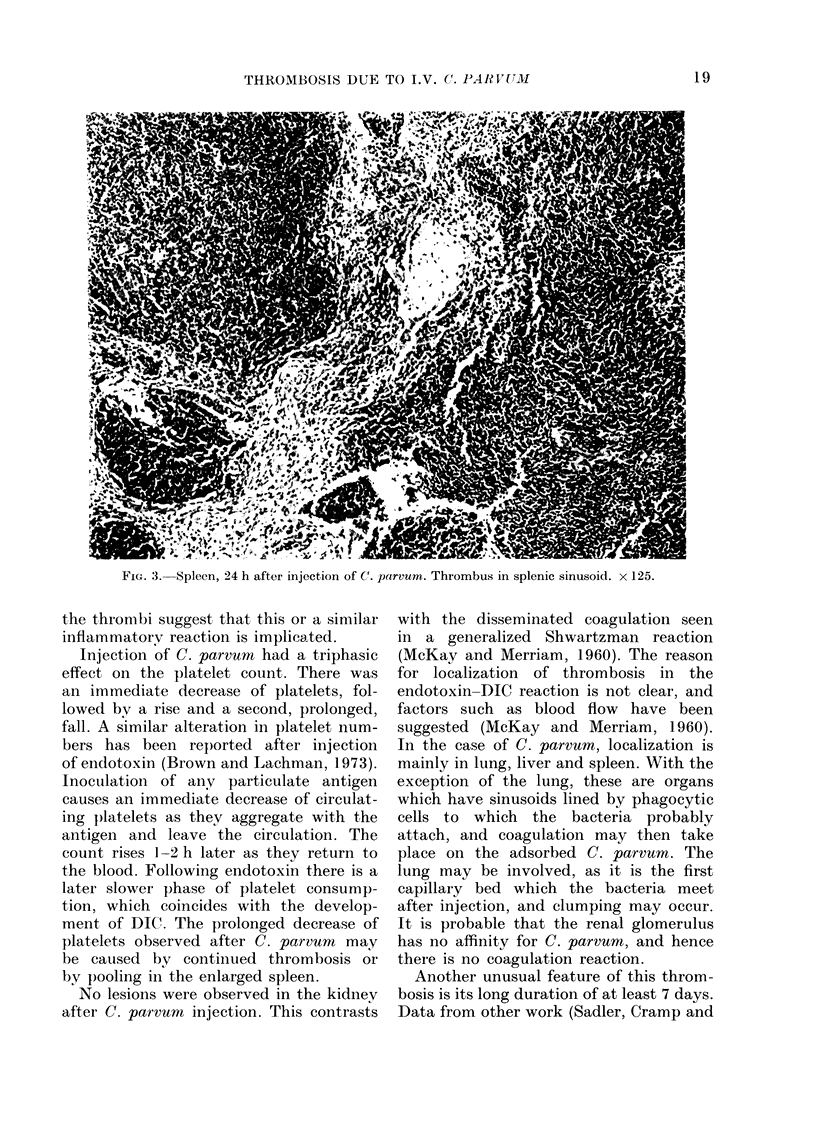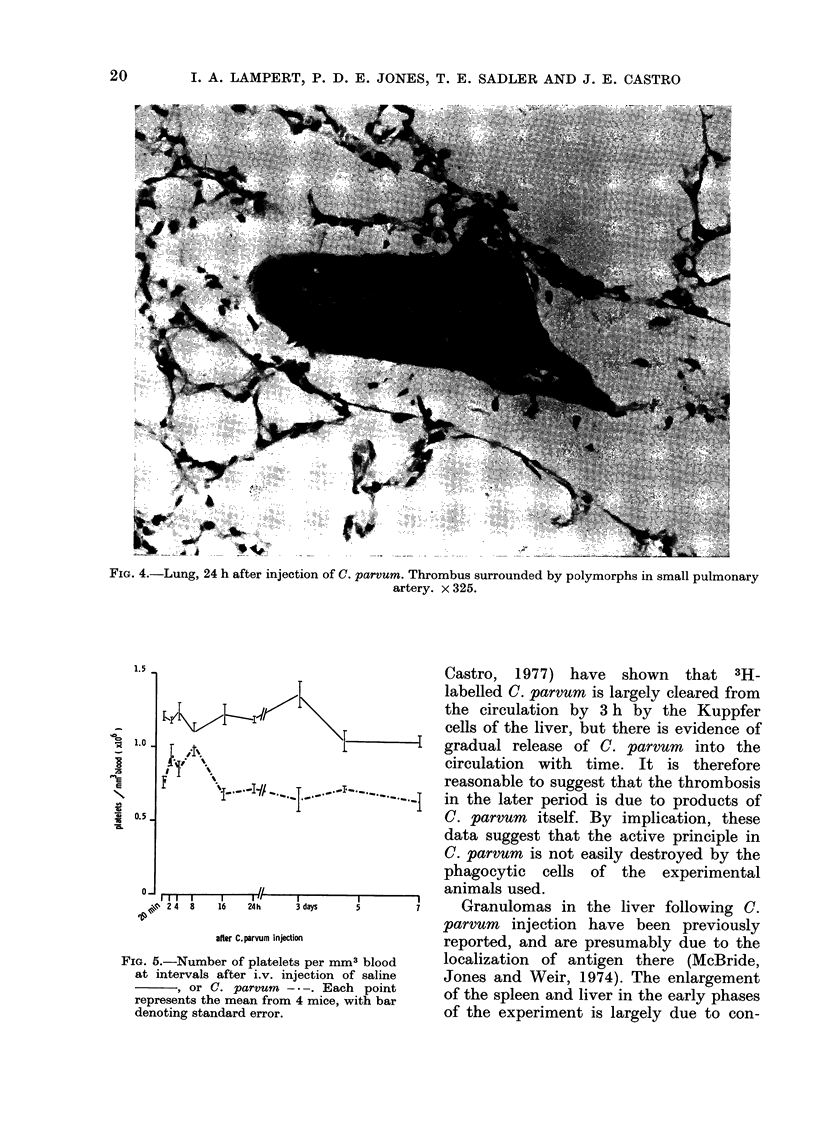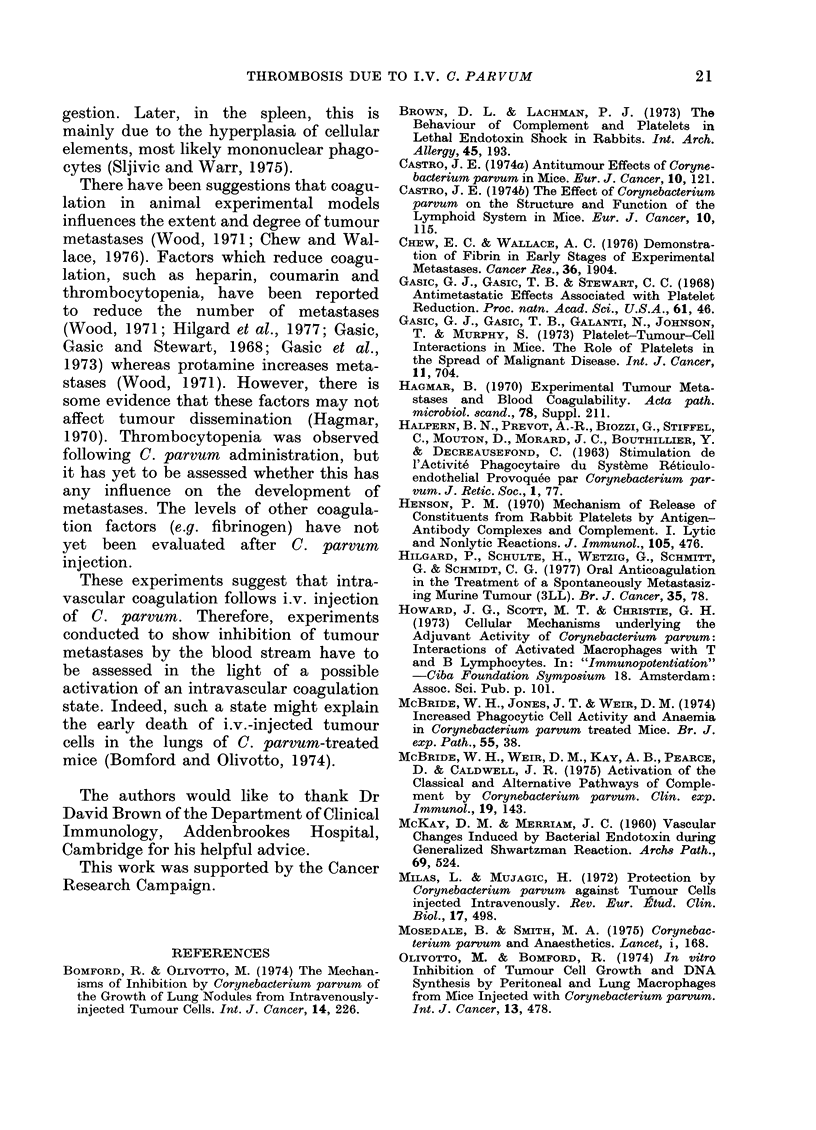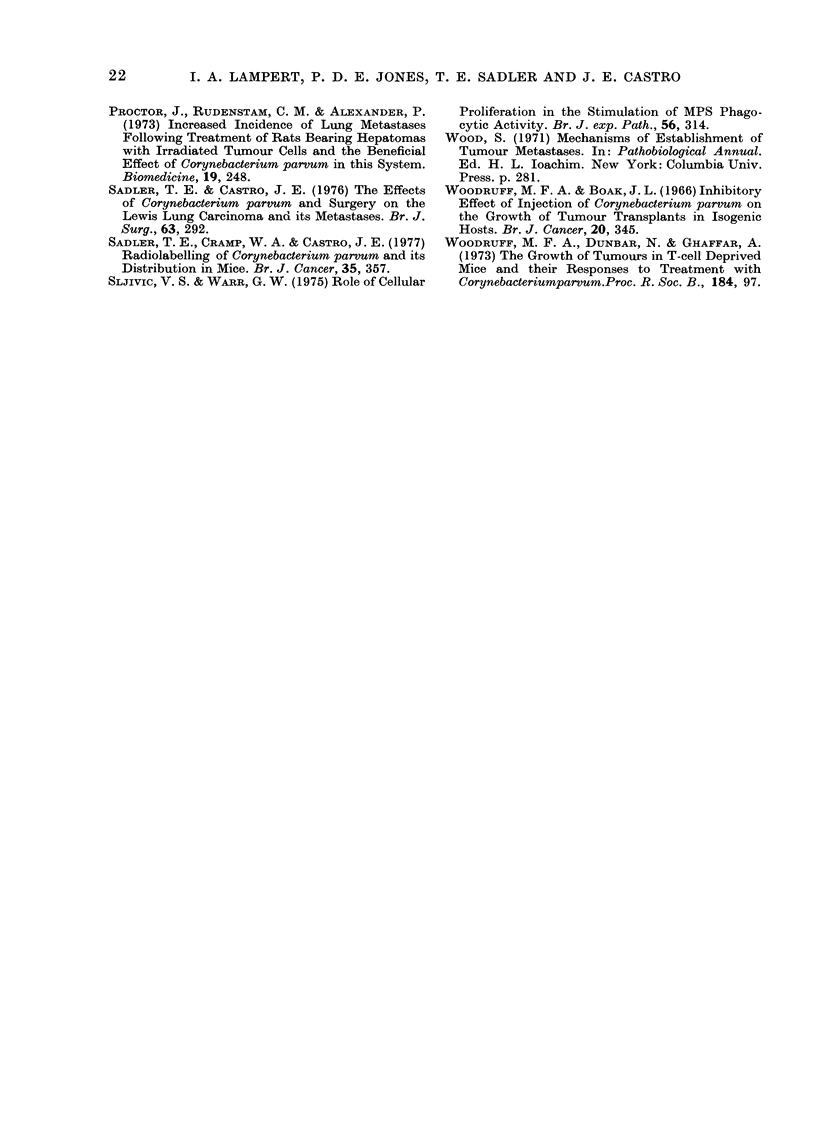# Intravascular coagulation resulting from intravenous injection of C. parvum in mice.

**DOI:** 10.1038/bjc.1977.149

**Published:** 1977-07

**Authors:** I. A. Lampert, P. D. Jones, T. E. Sadler, J. E. Castro

## Abstract

**Images:**


*Br. J. Cancer* (1977) **36**, 15

### INTRAVASCULAR COAGULATION RESULTING FROM INTRAVENOUS INJECTION OF *C. PARVUM* IN MICE

I. A. LAMPERT, P. D. E. JONES, T. E. SADLER and J. E. CASTRO

*From the Departments of Histopathology and Surgery, Royal Postgraduate Medical School, Hammersmith Hospital, London W12 0HS*

Received 15 December 1976  Accepted 28 February 1977

**Summary.—In mice, i.v. *C. parvum* induces intravascular coagulation. This is a prolonged reaction lasting up to 7 days. It results in thrombosis in hepatic vessels with consequent hepatic necrosis, and thrombosis in pulmonary and splenic vessels. This may be important in the assessment of the tumour-inhibitory activity of *C. parvum*.**

IN RODENTS, administration of killed *Corynebacterium parvum* decreases the growth of both subcutaneous and ascitic syngeneic tumours (Woodruff and Boak, 1966; Castro, 1974*a*). It also inhibits development of tumour nodules in the lung due either to i.v. injection of tumour cells (Milas and Mujagic, 1972; Bomford and Olivotto, 1974) or spontaneous metastases (Proctor, Rudenstam andAlexander, 1973; Sadler and Castro, 1976).

The mechanism of this anti-tumour action is under investigation. However, as *C. parvum* is a known immunopotentiating agent (Halpern *et al.*, 1963; Howard, Scott and Christie, 1973), most emphasis has been on the role played by macrophages and T cells (Woodruff, Dunbar and Ghaffar, 1973; Olivotto and Bomford, 1974). In this paper we present evidence of a non-immunological action of *C. parvum* which could possibly inhibit tumours, particularly metastases, in the liver and lungs.

This investigation stems from the observations that mice treated with i.v. *C. parvum* show macroscopic abnormalities of their livers (Castro, 1974*b*; Mosedale and Smith, 1975) and are abnormally sensitive to barbiturates (which are metabolized in liver tissue). A histological study was, therefore, made of the liver and other tissues of mice at intervals after injection of *C. parvum*.

### MATERIALS AND METHODS

*Mice.*—Age-matched, female C57BL/10 ScSn mice weighing 18–23 g were obtained from Olac (Southern) Ltd.

*C. parvum.*—A formalin-killed suspension of *C. parvum* (Wellcome strain CN6134, Batch BA 3935/A, 7 mg dry wt/ml) was injected i.v. at a dose of 0·466 mg in 0·2 ml normal saline. In one experiment, mice were given 1/10, 1/100 or 1/1000 of this dose. Control mice received an equal volume of saline or 0·01% thiomersalate (the preservative used for the *C. parvum*).

*Histology.*—Mice were killed by cervical dislocation in groups of 3 at 20 min, 4, 8, 16 and 24 h, and 3, 5 and 7 days after injection. Control mice, and those given reduced doses of *C. parvum*, were killed on Days 1 and 3 only. The following tissues were removed: liver, spleen, kidney, a segment of small gut, heart, mesenteric lymph nodes and thymus. The lungs were inflated *in situ* with formol-buffered saline and removed. All tissues were fixed in formol-buffered saline, processed in paraffin wax, sectioned at 7 μm and stained with haematoxylin and eosin.

*Platelet count.*—Groups of 4 mice were given i.v. *C. parvum* and blood was sampled at 20 min, 4, 8, 16 and 24 h, and 3, 5 and 7 days after injection. Bleeding was induced from the retro-orbital plexus using a heparinized capillary tube (Hawksley, England), and a 0·02-ml sample of the effusing blood was immediately collected into a heparinized white-backed micro-pipette. The 0·02-ml sample was diluted in 2 ml of 1% ammonium oxalate, in a plastic microcapped tube, and was mechanically shaken for 3 to 5 min. A Neubauer counting chamber was filled with the diluted sample and left for 15 to 20 min in a moist container in order to allow the platelets to settle. Platelets were then counted under phase, using a ×10 objective and the mean total count (±s.e.) estimated.

### RESULTS

Within 10 to 20 min after i.v. injection of 0·466 mg *C. parvum*, C57BL mice were observed to be in respiratory distress. They became cold to the touch and were "hunched over". This syndrome disappeared after 2 h.

Groups of 3 mice were killed at intervals after injection. Some necrosis was observed macroscopically in the liver as early as 24 h. By 7 days all of the livers were enlarged, pale and mottled with necrotic patches. The mean weight of the whole body, and various tissues are shown in the Table. There was a 5-fold increase in spleen weight, and a doubling of liver weight.

### *Histology*

Twenty minutes after injection, occasional thrombi surrounded by polymorphs were seen in the sinusoids of the liver and in the alveolar capillaries: a minority showed polymorphs aggregated about their periphery. At 4 h, all mice showed numerous aggregates of polymorphs surrounding fibrin thrombi in the sinusoids of the liver, and a few similar thrombi in the hepatic and portal veins. At 8 h, the number of these venous thrombi surrounded by polymorphs was increased. In addition, thrombi were seen in the splenic pulp sinusoids. At 16 h, infarcts, without significant inflammatory response in their periphery, were seen in the liver, and the numbers of pulmonary and splenic thrombi were increased, with consequent congestion of the splenic pulp. These changes were further increased at 24 h, the hepatic infarcts (see Figs. 1 and 2) were concentrated at the hepatic capsule and were also present elsewhere in the parenchyma. When observed on the periphery of the liver, they were roughly triangular in shape, with the broad base of the triangle on the capsule; frequently there was a thrombosed vessel at the apex. At this stage, a predominantly macrophage response was seen at the edge of the infarcted area.

The number and size of thrombi were increased in the splenic pulp and in the pulmonary vessels (see Figs. 3 and 4). Few thrombi were seen in the medullary vessels of the lymph nodes.

By Day 3, thrombi in the hepatic vessels were showing organization, and were infiltrated by phagocytic cells. A

TABLE.—*Weights (Mean ± s.d.) of Whole Body and Various Tissues of Groups of* 3 *Mice at Intervals after i.v. Injection of* C. parvum

| Time | Whole body (g) | Liver (mg) | Spleen (mg) | Kidney (mg) | Mes* LN (mg) | Thymus (mg) |
|---|---|---|---|---|---|---|
| Control | 20·0±0·9 | 914±55 | 85±13 | 115± 5 | 30·0± 3·0 | 50·0±10·4 |
| 20 min | 20·8±1·9 | 971±52 | 85±13 | 109±13 | 34·8± 2·1 | 66·2± 9·5 |
| 4 h | 20·8±0·8 | 1124±327 | 102±44 | 113± 9 | 30·7± 2·3 | 56·0± 9·6 |
| 8 h | 20·3±1·0 | 912±63 | 89± 2 | 115± 9 | 36·7± 3·5 | 54·7±18·3 |
| 16 h | 19·8±0·8 | 1065±93 | 115± 5 | 110± 1 | 31·3± 3·2 | 58·5± 8·2 |
| 24 h | 19·5±0·5 | 1056±64 | 113± 8 | 115± 7 | 35·7± 3·4 | 49·3± 9·8 |
| 3 Day | 19·8±0·8 | 1189±131 | 197±15 | 120± 8 | 34·3± 2·8 | 45·5± 5·5 |
| 5 Day | 21·0±0·3 | 1463±62 | 233±33 | 141±12 | 36·5± 6·5 | 48·2±12·3 |
| 7 Day | 20·7±1·5 | 1930±31 | 431±41 | 142± 5 | 29·0±10·6 | 35·8±11·6 |

* Mesenteric lymph nodes.

FIG. 1.—Liver, 24 h after injection of *C. parvum*. Thrombus in portal vein surrounded by polymorphs. × 125.

similar infiltrate was seen at the periphery of the liver infarcts, with a partial removal of necrotic tissue. At this stage, numerous macrophage granulomas were seen in the hepatic parenchyma. The thrombi in the pulmonary and splenic vessels showed organization similar to that seen in the liver. The splenic red pulp was increased in size both by congestion and by an increase in the number of mononuclear cells.

At 5 days, the liver showed numerous small macrophage granulomas. Large collections of loosely aggregated macrophages marked the position of removed hepatic parenchyma. Thrombi, surrounded by polymorphs and not showing any form of organization, were seen in hepatic vessels. These were, therefore, similar to those observed in the first 24 h. Also, infarcts were seen in the hepatic parenchyma, showing absent or incomplete karyorrhexis and no inflammatory reaction. New thrombi, similar to those seen in the liver, were seen in the pulmonary and splenic vessels. The spleen also showed a marked increase in the number of megakaryocytes in the red pulp. By 7 days there was extensive replacement of the hepatic parenchyma by granulomas, and necrotic areas of liver. The surviving parenchymal cells showed numerous mitoses. Occasional megakaryocytes were seen in the liver sinusoids.

Throughout the experiment, no abnormality was seen in the kidneys, heart, small gut or thymus. After injection of a 1/10 dilution of *C. parvum*, occasional granulomas were seen in the liver, and thrombi in the pulmonary vessels. At higher dilutions (1/100 and 1/1000) these became fewer.

No abnormalities were observed in the tissues of mice which had received saline or thiomersalate.

FIG. 2.—Liver, 24 h after injection of *C. parvum*. Thrombus in hepatic vein. Infarction of liver. ×125.

*Platelet counts*

There was an initial fall in platelet count as early as 20 min after the injection of *C. parvum* (Fig. 5). Subsequently, there was an increase, reaching a maximum at 8 h. This was followed by a second fall to 50% of the control value by 16 h. This low level remained constant for 7 days, and did not return towards normal until 21 days after injection.

### DISCUSSION

At the high dose used, *C. parvum* induced widespread intravascular thrombosis. The organ most affected was the liver, with significant effects in the spleen and lung. Thrombosis was mirrored by a fall in the platelet counts. Ageing of the thrombi was assessed by their organization. Consequently the presence of thrombi in vessels surrounded by polymorphs at 5 and 7 days suggested that these were fresh thrombi. Further evidence that thrombosis was continuing was the continued low platelet count, despite evidence of megakaryocytic hyperplasia. The presence of fresh infarcts in the liver attest to the importance of these thrombi.

The mechanism of this reaction is at the moment a matter for speculation. By analogy with the disseminated intravascular coagulation (DIC) reaction caused by endotoxin, the following mechanism could be suggested. *C. parvum*, like other particulate antigens, is known to activate the alternate complement pathway (McBride *et al.*, 1975). In endotoxin shock (Brown and Lachman, 1973) activated C3 shows immune adherence to the platelets of many non-primate species, including mice (Henson, 1970). This results in disruption of platelets, with the release of factors which produce thrombosis. The presence of polymorphs surrounding

FIG. 3.—Spleen, 24 h after injection of *C. parvum*. Thrombus in splenic sinusoid. ×125.

the thrombi suggest that this or a similar inflammatory reaction is implicated.

Injection of *C. parvum* had a triphasic effect on the platelet count. There was an immediate decrease of platelets, followed by a rise and a second, prolonged, fall. A similar alteration in platelet numbers has been reported after injection of endotoxin (Brown and Lachman, 1973). Inoculation of any particulate antigen causes an immediate decrease of circulating platelets as they aggregate with the antigen and leave the circulation. The count rises 1–2 h later as they return to the blood. Following endotoxin there is a later slower phase of platelet consumption, which coincides with the development of DIC. The prolonged decrease of platelets observed after *C. parvum* may be caused by continued thrombosis or by pooling in the enlarged spleen.

No lesions were observed in the kidney after *C. parvum* injection. This contrasts with the disseminated coagulation seen in a generalized Shwartzman reaction (McKay and Merriam, 1960). The reason for localization of thrombosis in the endotoxin–DIC reaction is not clear, and factors such as blood flow have been suggested (McKay and Merriam, 1960). In the case of *C. parvum*, localization is mainly in lung, liver and spleen. With the exception of the lung, these are organs which have sinusoids lined by phagocytic cells to which the bacteria probably attach, and coagulation may then take place on the adsorbed *C. parvum*. The lung may be involved, as it is the first capillary bed which the bacteria meet after injection, and clumping may occur. It is probable that the renal glomerulus has no affinity for *C. parvum*, and hence there is no coagulation reaction.

Another unusual feature of this thrombosis is its long duration of at least 7 days. Data from other work (Sadler, Cramp and

FIG. 4.—Lung, 24 h after injection of *C. parvum*. Thrombus surrounded by polymorphs in small pulmonary artery. ×325.

FIG. 5.—Number of platelets per mm³ blood at intervals after i.v. injection of saline ———, or *C. parvum* —·—. Each point represents the mean from 4 mice, with bar denoting standard error.

Castro, 1977) have shown that ³H-labelled *C. parvum* is largely cleared from the circulation by 3 h by the Kuppfer cells of the liver, but there is evidence of gradual release of *C. parvum* into the circulation with time. It is therefore reasonable to suggest that the thrombosis in the later period is due to products of *C. parvum* itself. By implication, these data suggest that the active principle in *C. parvum* is not easily destroyed by the phagocytic cells of the experimental animals used.

Granulomas in the liver following *C. parvum* injection have been previously reported, and are presumably due to the localization of antigen there (McBride, Jones and Weir, 1974). The enlargement of the spleen and liver in the early phases of the experiment is largely due to congestion. Later, in the spleen, this is mainly due to the hyperplasia of cellular elements, most likely mononuclear phagocytes (Sljivic and Warr, 1975).

There have been suggestions that coagulation in animal experimental models influences the extent and degree of tumour metastases (Wood, 1971; Chew and Wallace, 1976). Factors which reduce coagulation, such as heparin, coumarin and thrombocytopenia, have been reported to reduce the number of metastases (Wood, 1971; Hilgard *et al.*, 1977; Gasic, Gasic and Stewart, 1968; Gasic *et al.*, 1973) whereas protamine increases metastases (Wood, 1971). However, there is some evidence that these factors may not affect tumour dissemination (Hagmar, 1970). Thrombocytopenia was observed following *C. parvum* administration, but it has yet to be assessed whether this has any influence on the development of metastases. The levels of other coagulation factors (*e.g.* fibrinogen) have not yet been evaluated after *C. parvum* injection.

These experiments suggest that intravascular coagulation follows i.v. injection of *C. parvum*. Therefore, experiments conducted to show inhibition of tumour metastases by the blood stream have to be assessed in the light of a possible activation of an intravascular coagulation state. Indeed, such a state might explain the early death of i.v.-injected tumour cells in the lungs of *C. parvum*-treated mice (Bomford and Olivotto, 1974).

The authors would like to thank Dr David Brown of the Department of Clinical Immunology, Addenbrookes Hospital, Cambridge for his helpful advice.

This work was supported by the Cancer Research Campaign.

### REFERENCES

Bomford, R. & Olivotto, M. (1974) The Mechanisms of Inhibition by *Corynebacterium parvum* of the Growth of Lung Nodules from Intravenously-injected Tumour Cells. *Int. J. Cancer*, **14**, 226.

Brown, D. L. & Lachman, P. J. (1973) The Behaviour of Complement and Platelets in Lethal Endotoxin Shock in Rabbits. *Int. Arch. Allergy*, **45**, 193.

Castro, J. E. (1974*a*) Antitumour Effects of *Corynebacterium parvum* in Mice. *Eur. J. Cancer*, **10**, 121.

Castro, J. E. (1974*b*) The Effect of *Corynebacterium parvum* on the Structure and Function of the Lymphoid System in Mice. *Eur. J. Cancer*, **10**, 115.

Chew, E. C. & Wallace, A. C. (1976) Demonstration of Fibrin in Early Stages of Experimental Metastases. *Cancer Res.*, **36**, 1904.

Gasic, G. J., Gasic, T. B. & Stewart, C. C. (1968) Antimetastatic Effects Associated with Platelet Reduction. *Proc. natn. Acad. Sci., U.S.A.*, **61**, 46.

Gasic, G. J., Gasic, T. B., Galanti, N., Johnson, T. & Murphy, S. (1973) Platelet–Tumour–Cell Interactions in Mice. The Role of Platelets in the Spread of Malignant Disease. *Int. J. Cancer*, **11**, 704.

Hagmar, B. (1970) Experimental Tumour Metastases and Blood Coagulability. *Acta path. microbiol. scand.*, **78**, Suppl. 211.

Halpern, B. N., Prevot, A.-R., Biozzi, G., Stiffel, C., Mouton, D., Morard, J. C., Bouthillier, Y. & Decreausefond, C. (1963) Stimulation de l'Activité Phagocytaire du Système Réticuloendothelial Provoquée par *Corynebacterium parvum*. *J. Retic. Soc.*, **1**, 77.

Henson, P. M. (1970) Mechanism of Release of Constituents from Rabbit Platelets by Antigen–Antibody Complexes and Complement. I. Lytic and Nonlytic Reactions. *J. Immunol.*, **105**, 476.

Hilgard, P., Schulte, H., Wetzig, G., Schmitt, G. & Schmidt, C. G. (1977) Oral Anticoagulation in the Treatment of a Spontaneously Metastasizing Murine Tumour (3LL). *Br. J. Cancer*, **35**, 78.

Howard, J. G., Scott, M. T. & Christie, G. H. (1973) Cellular Mechanisms underlying the Adjuvant Activity of *Corynebacterium parvum*: Interactions of Activated Macrophages with T and B Lymphocytes. In: "*Immunopotentiation*" —*Ciba Foundation Symposium* 18. Amsterdam: Assoc. Sci. Pub. p. 101.

McBride, W. H., Jones, J. T. & Weir, D. M. (1974) Increased Phagocytic Cell Activity and Anaemia in *Corynebacterium parvum* treated Mice. *Br. J. exp. Path.*, **55**, 38.

McBride, W. H., Weir, D. M., Kay, A. B., Pearce, D. & Caldwell, J. R. (1975) Activation of the Classical and Alternative Pathways of Complement by *Corynebacterium parvum*. *Clin. exp. Immunol.*, **19**, 143.

McKay, D. M. & Merriam, J. C. (1960) Vascular Changes Induced by Bacterial Endotoxin during Generalized Shwartzman Reaction. *Archs Path.*, **69**, 524.

Milas, L. & Mujagic, H. (1972) Protection by *Corynebacterium parvum* against Tumour Cells injected Intravenously. *Rev. Eur. Étud. Clin. Biol.*, **17**, 498.

Mosedale, B. & Smith, M. A. (1975) *Corynebacterium parvum* and Anaesthetics. *Lancet*, i, 168.

Olivotto, M. & Bomford, R. (1974) *In vitro* Inhibition of Tumour Cell Growth and DNA Synthesis by Peritoneal and Lung Macrophages from Mice Injected with *Corynebacterium parvum*. *Int. J. Cancer*, **13**, 478.

PROCTOR, J., RUDENSTAM, C. M. & ALEXANDER, P. (1973) Increased Incidence of Lung Metastases Following Treatment of Rats Bearing Hepatomas with Irradiated Tumour Cells and the Beneficial Effect of *Corynebacterium parvum* in this System. *Biomedicine*, **19**, 248.

SADLER, T. E. & CASTRO, J. E. (1976) The Effects of *Corynebacterium parvum* and Surgery on the Lewis Lung Carcinoma and its Metastases. *Br. J. Surg.*, **63**, 292.

SADLER, T. E., CRAMP, W. A. & CASTRO, J. E. (1977) Radiolabelling of *Corynebacterium parvum* and its Distribution in Mice. *Br. J. Cancer*, **35**, 357.

SLJIVIC, V. S. & WARR, G. W. (1975) Role of Cellular Proliferation in the Stimulation of MPS Phagocytic Activity. *Br. J. exp. Path.*, **56**, 314.

WOOD, S. (1971) Mechanisms of Establishment of Tumour Metastases. In: *Pathobiological Annual.* Ed. H. L. Ioachim. New York: Columbia Univ. Press. p. 281.

WOODRUFF, M. F. A. & BOAK, J. L. (1966) Inhibitory Effect of Injection of *Corynebacterium parvum* on the Growth of Tumour Transplants in Isogenic Hosts. *Br. J. Cancer*, **20**, 345.

WOODRUFF, M. F. A., DUNBAR, N. & GHAFFAR, A. (1973) The Growth of Tumours in T-cell Deprived Mice and their Responses to Treatment with *Corynebacteriumparvum.Proc. R. Soc. B.*, **184**, 97.